# In-Cold Exposure to Z-3-Hexenal Provides Protection Against Ongoing Cold Stress in *Zea mays*

**DOI:** 10.3390/plants8060165

**Published:** 2019-06-11

**Authors:** Marie Engelberth, Samantha M. Selman, Jurgen Engelberth

**Affiliations:** Department of Biology, University of Texas at San Antonio, One UTSA Circle, San Antonio, TX 78249, USA; jengelberth@gmx.net (M.E.); sammiesprinkles27@gmail.com (S.M.S.)

**Keywords:** green leaf volatiles, *Zea mays*, Z-3-hexenal, abiotic stress, cold stress, ion leakage, cold protection

## Abstract

Green leaf volatiles (GLVs), which have mostly been described as providers of protection against insect herbivory and necrotrophic pathogen infections, were recently shown to provide significant fortification against cold stress damage. It was further demonstrated that cold-damaged maize seedlings released a significant amount of GLVs, in particular, Z-3-hexenal (Z-3-HAL). Here, we report that the in-cold treatment of maize seedlings with Z-3-HAL significantly improved cold stress resistance. The transcripts for cold-protective genes were also significantly increased in the Z-3-HAL treated maize seedlings over those found in only cold stressed plants. Consequently, the maize seedlings treated with HAL during cold showed a significantly increased structural integrity, significantly less damage, and increased growth after cold stress, relative to the non-HAL treated maize seedlings. Together, these results demonstrate the protective effect of in-cold treatment with HAL against cold damage, and suggest that the perception of these compounds during cold episodes significantly improves resistance against this abiotic stress.

## 1. Introduction

Plant volatiles have emerged as an ecologically significant means used by plants to communicate various stresses, not only to distant parts of the affected plants, but also to other plants nearby. While hormone-based signaling pathways regulate the bulk of the responses, plant volatiles often provide additional protection either by directly activating sections of the respective stress response, or by priming these responses, resulting in a faster and/or stronger response. Recently, we found that green leaf volatiles (GLVs) provide such protection, not only against insect herbivory, but also against cold stress damage [1].

GLVs are a blend of fatty acid derived compounds, which are typically associated with damage to photosynthetic tissues, and are responsible for the characteristic “green” smell of plants [2,3,4,5]. The biosynthesis of GLVs is well understood, and begins with the regiospecific dioxygenation of α-linolenic- or linoleic-acid by a *13-lipoxygenase (13-LOX)* to form 13(*S*)-hydroperoxy-9(*Z*), 11(*E*), 15(*Z*)-octadecatrienoic acid (13-HPOT), 13(*S*)-hydroperoxy-9(*Z*), or 11(*E*)-octadecadienoic acid (13-HPOD), respectively. These 13(*S*)-hydroperoxy acids are then cleaved by hydroperoxide lyase (HPL) to produce either Z-3-HAL from 13-HPOT, or hexanal from 13-HPOD. Z-3-HAL may then be reduced to form (*Z*)-3-hexen-1-ol (Z-3-HOL) by a NADPH-dependent reductase [6] in intact cells, which can then further be converted to (*Z*)-3-hexeny-1-yl acetate (Z-3-HAC) by acetyl-CoA:(*Z*)-3-hexen-1-ol acetyltransferase [7], a step that is also performed by intact cells. In some plants, Z-3-HAL may become converted to (*E*)-2-hexen-1-al, either spontaneously, or enzymatically by a (*Z*)-3:(*E*)-2-hexenal isomerase [8,9]. GLVs are released almost instantly from damaged leaves [2,3,6], with Z-3-HAL being the major product. However, some GLVs can also be released from undamaged plants, mainly in the form of Z-3-HAC, at the onset of darkness [10].

GLVs have mainly been described to play a role in mediating plant–insect interactions. Specifically, they were found to prime plants against insect herbivory by inducing a faster and/or stronger response during an attack. This was first reported in maize by Engelberth et al. [11], but has since been confirmed for other plant species, including *Nicotiana attenuata* [12], *Phaseolus lunatus* L. [13], and *Populus deltoides* × *nigra* [14]. Although the defensive role of GLVs is now well established, a functional role for GLVs in regulating abiotic stresses is only beginning to emerge, and it was often limited to the analysis of their emission [15,16]. Recently however, Yamauchi et al. [17] demonstrated that the GLV (*E*)-2-hexen-1-al, together with other reactive short-chain volatile compounds, induced abiotic stress-related genes in *Arabidopsis,* including an essential transcription factor, *DREB2A,* involved in the regulation of drought responsive genes. Interestingly, other GLVs, including Z-3-HAL or Z-3-HOL, were not active. 

We recently identified multiple GLV-responsive genes in maize [18], including several that are normally induced during cold and drought stress [1]. These included dehydrins (DEH1, gene model at maizegdb.org GRMZM2G373522; DEH2, GRMZM2G147014), a proteolipid (PROT, GRMZM2G015605), as well as adhesive/proline-rich proteins (APRP1, GRMZM2G003937; APRP2, GRMZM2G066197_P02), low temperature inducible proteins (LTI, GRMZM2G181551), and late embryogenesis abundant proteins (LEP1, GRMZM2G352415; LEP2, GRMZM2G045664). As members of the cold regulatory complex, these gene products have previously been shown to provide protection by maintaining cellular integrity in cold and drought stressed plants, resulting in significantly reduced damage [19,20,21,22,23,24,25,26]. This implied that the activation of these cold regulated (COR) genes by GLVs may provide additional protection against these stresses, and we actually found that the treatment of maize seedlings with Z-3-HAC provided significant protection against cold stress damage [1]. In the same study, we found that cold-damaged corn seedlings, in turn, also released significant amounts of GLVs, in particular, Z-3-HAL. However, while the damage was inflicted by cold stress, the release of GLVs was measured at room temperature (RT). This raised the question of whether GLV will also be released under damaging cold stress, and if so, would they provide protection under those conditions. To answer these questions, we performed experiments designed to test for the biological activities of GLVs under cold stress conditions. We found that in-cold treatment with Z-3-HAL, the major GLV released under cold damaging conditions, stimulated transcript accumulation for selected COR genes, provided significant protection against cold damage, and stimulated growth after a cold episode. To our knowledge, this is the first report on GLV-mediated protection under these conditions, and it sheds new light on the potential of GLVs to provide additional protection, even within an ongoing stress situation.

## 2. Results

### 2.1. In-Cold Release of GLVs

**Figure 1:**
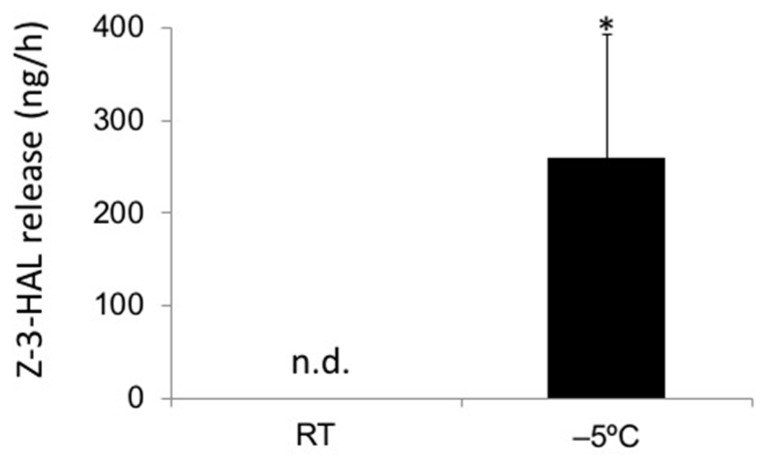
Release of green leaf volatiles from cold stress damaged maize (*Zea mays*) seedlings. Maize seedlings were either placed at −5 °C or at room temperature (RT), and volatiles were collected for 1 h (*n* = 4). Values are means ± standard deviation (SD), and asterisks indicate significant differences between treated and untreated seedlings (*t*-test, *p* < 0.05; n.d., not detectable).

Nearly all plants release GLVs when mechanically damaged. However, most if not all of these studies were performed at RT. As cold stress can also inflict significant damage, we wanted to test if plants damaged by cold stress also release significant amounts of these compounds. To ensure damage, we placed maize seedlings in a chamber at −5 °C for 1 h, and collected volatiles over this time. The controls were kept at RT (26 °C). Over the collection period, we found that cold damaged maize seedlings released on average 258.1 ± 135.1 ng Z-3-HAL per hour per plant (Figure 1). We were not able to detect any significant amount of other GLVs under those conditions. This clearly shows that even during severe cold, the plants that sustained significant damage may still release substantial amounts of GLVs, in particular Z-3-HAL, making cold-stressed plants very capable of signaling damage to other plants nearby.

### 2.2. Z-3-HAL-Responsive Gene Expression during In-Cold Treatment

**Figure 2:**
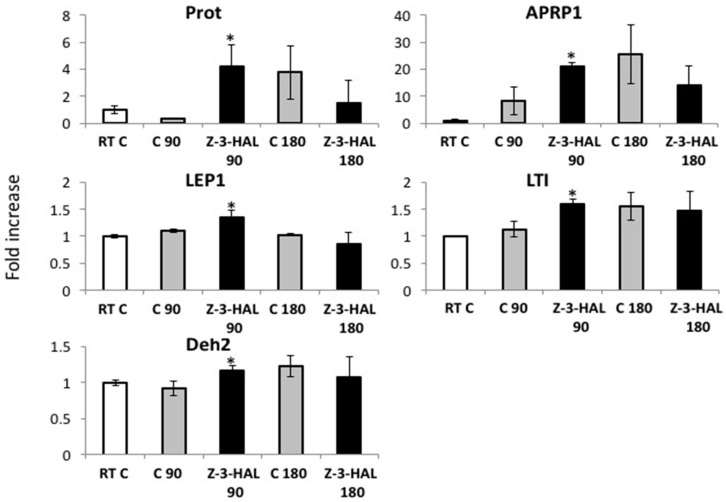
The effect of in-cold (*Z*)-3-hexen-1-al (Z-3-HAL) treatment on cold stress related gene expression in maize (*Zea mays*) seedlings (*n* = 3) under cold stress (5 °C). Seedlings were treated with physiological concentrations of Z-3-HAC (20 μg L^−1^) 30 min after being placed at 5 °C for 90 min and 180 min. Transcript levels were measured by RT-qPCR. The values are means ± standard error of the mean (SEM), and the asterisks indicate significant differences between the treated and untreated seedlings (*t*-test, *p* < 0.05).

Based on our finding that cold-damaged maize seedlings almost exclusively release Z-3-HAL upon cold stress damage, we focused our experiments on the effects of this particular GLV, and how it may affect maize seedlings when treated with under cold stress conditions. We first tested if the treatment of maize seedlings that were already exposed to cold stress with Z-3-HAL had any effect on cold stress-related gene expression. We found that the transcripts for typical *COR* genes, like *APRP1*, *DEH2*, *LEP1*, *PROT*, and *LTI,* accumulated at significantly higher levels in maize seedlings treated with 20 μg of Z-3-HAL 90 min after being added to the cold treated maize seedlings, ranging from a 12-fold increase of PROT to a 1.21-fold increase for LEP1 (Figure 2).

At this time point, the maize seedlings were already 120 min at 4 °C. There was no significant difference in the transcript levels of all of the selected genes 180 min after Z-3-HAL (210 min at 4 °C), however, the Z-3-HAL-treated maize seedlings appear to show a tendency for an earlier reduction in transcript accumulation than their respective controls.

These results clearly demonstrate that the physiological concentrations of Z-3-HAL, when used during the cold, can induce significant levels of transcripts of *COR* genes early on, thereby providing the first evidence that maize seedlings respond to this volatile compound, even under those conditions. Furthermore, it strongly suggests that the perception of Z-3-HAL during the cold may add to the fortification of plants against severe cold damage.

### 2.3. Conductivity Assays and Damage Assessment Revealed Significant Increase in Structural Integrity and Damage Protection during Cold Stress

**Figure 3:**
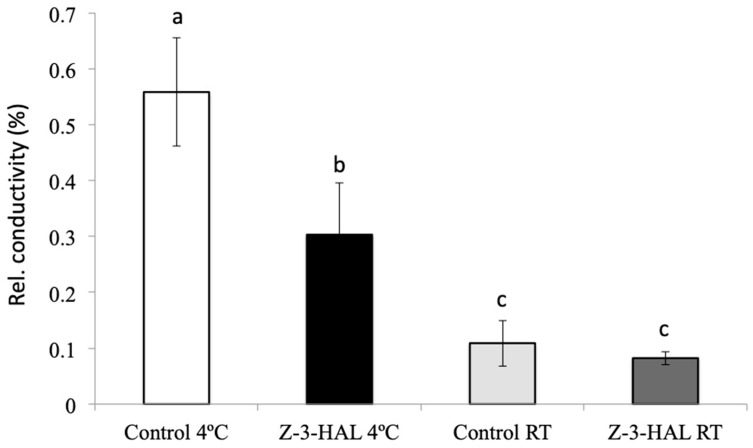
The effect of in-cold (*Z*)-3-hexen-1-al (Z-3-HAL) treatment on cold stress related ion leakage in maize (*Zea mays*) seedlings (*n* = 6). The relative conductivity is expressed as the ratio between ion leakage after short cold stress, divided by the total ion leakage after overnight freezing and thawing. The values are means ± SD, and different letters indicate significant differences between the treated and untreated seedlings (ANOVA, *p* < 0.05).

Many of the *COR* genes analyzed for this study have previously been shown to stabilize cellular structures against damage from cold stress. Conductivity assays were therefore performed in order to establish a functioning role for in-cold Z-3-HAL treatment to increase the structural integrity in the leaves of maize seedlings. We found that the maize seedlings treated in-cold with Z-3-HAL had significantly less ion leakage when compared to their only cold-treated controls (30% ion leakage), compared to 55% relative ion leakage in cold-only-treated leaf discs. The control leaf discs for both treatments that were kept at RT, and were also found to differ in their ion leakage. For in-cold Z-3-HAL-treated leaf discs kept at RT, we found an 8% leakage, while for the control leaf discs, an 11% leakage was determined (Figure 3). These results clearly demonstrate a significant improvement of structural integrity for in-cold Z-3-HAL-treated maize seedlings over their respective controls, and support our hypothesis that in-cold treatment with Z-3-HAL adds significant protection.

**Figure 4:**
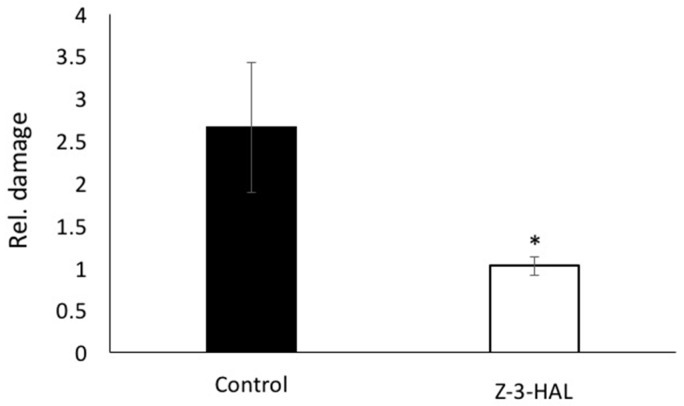
The effect of in-cold (*Z*)-3-hexen-1-al (Z-3-HAL) treatment on damage in maize (*Zea mays*) seedlings. Assessment of damage on a scale from 0 (no damage) to 4 (dead). The values are means ± SD, and the asterisks indicate significant differences between the treated and untreated seedlings (Students *t*-test, *p* < 0.05).

We further tested maize seedlings after in-cold treatment with Z-3-HAL for their resistance against cold stress damage, by assessing the damage after exposure to freezing temperatures (−5 °C) for 30 min. As both the control and Z-3-HAL-treated plants received cold treatment at 5 °C for 16 h before being transferred to the freezer, a cold acclimation was observed and the plants survived longer, without sustaining damage, than reported previously [1]. Therefore, while non-cold acclimated maize seedlings died after 15 min at −5 °C, cold acclimated plants were exposed to this temperature for 30 min. The plants were assessed based on our one (no damage) to five (dead) scale, by three individuals, independently (Figure 4). Among the Z-3-HAL acclimated maize seedling (eight plants) we found one leaf to have minor freeze damage, while all of the other leaves were undamaged, resulting in an average score of 1.02. The cold-acclimated control plants showed significantly more damage. One plant was dead, while all of the other plants showed at least damage to one leaf, or had broken leaves and/or stems, resulting in a total score of 2.7. The differences were found to be highly significant (*t*-test, *p* < 0.00001). Based on these findings, it can therefore be concluded that in-cold treatment with Z-3-HAL is highly effective, and provides significant protection against cold stress damage.

### 2.4. Increased Growth Response of In-Cold Z-3-HAL Treated Maize Seedlings after Cold Stress

**Figure 5:**
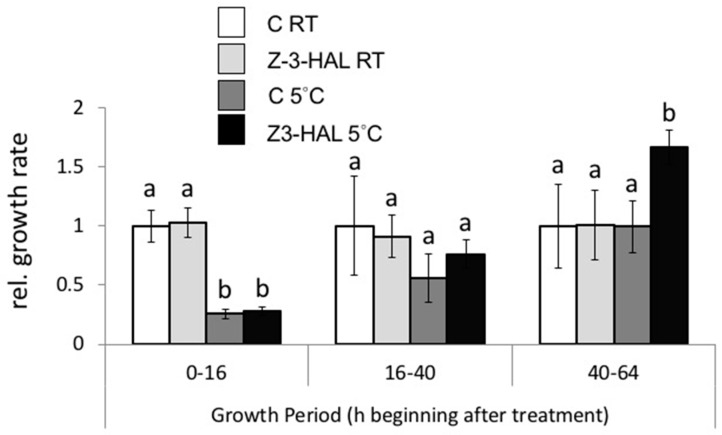
The growth response of in-cold (*Z*)-3-hexen-1-al (Z-3-HAL) and untreated maize (*Zea mays*) seedlings (*n* = 6) after 16 h at 5 °C and at room temperature (RT). Growth is expressed in relative units, with the RT control plants set at 100% for the observed growth period. The values are means ± SD, and the different letters indicate significant differences (ANOVA, *p* < 0.05).

We reported previously that maize seedlings treated with GLV showed an enhanced growth response after cold treatments. Here, we assessed the growth response after in-cold treatment with Z-3-HAL (Figure 5). Growth is expressed in relative units, with the RT control plants set at 100% for the observed growth period. As described previously [1], we found that the in-cold Z-3-HAL-treated and control cold seedlings displayed significantly reduced the growth rates over the first 16 h period of cold stress at 5 °C. when compared to the room temperature (RT) control plants (Figure 5). Both treatment groups only reached 26% to 27% of the RT control plant growth. Likewise, the control RT and the Z-3-HAL RT maize seedlings grew at similar rates over this period. The growth of the cold treated seedlings increased significantly between 16 h and 40 h after being removed from the cold, with the cold control seedlings reaching approximately 56% of the growth of the RT control seedlings, and the 5 °C + Z-3-HAL seedlings reaching nearly 76% of the growth rate of the RT control seedlings. Between 40 h and 64 h, the growth rate of the 5 °C control seedlings was again similar to the RT-control seedlings. However, the 5 °C + Z-3-HAL seedlings showed a significant increase in growth (166% of RT control) over this period. These data are in accordance with our previous results [1], and show that in-cold Z-3-HAL treatments also enhance seedling growth after overnight cold stress.

## 3. Discussion

Recently, we demonstrated that pre-treatment of maize seedlings with GLVs, in particular Z-3-HAC, provided better protection against cold stress damage, resulting in reduced damage and increased growth [1]. Mainly responsible for this protection was likely the activation of genes encoding cold regulated (COR) proteins, including *DEHs, LTIs, LEPs, PROTs, and APRPs*. Not only were these genes directly activated by the Z-3-HAC treatment, but their transcript accumulation during cold stress was also primed by a previous exposure to this GLV. However, Z-3-HAC is mainly produced by intact cells, while the damaged tissue mainly produces Z-3-HAL in the maize seedlings. As cold stress damage can cause a significant release of Z-3-HAL, as shown herein, we started to investigate a potential role for Z-3-HAL as a regulator of cold stress protection during a cold episode, at a time when the plants were already exposed to chilling and potentially damaging temperatures.

Cold stress is a major environmental stressor that often limits the growth, productivity, and distribution of plants [27,28,29]. Extended exposure to low temperatures negatively affects the membranes and membrane associated proteins, which, when damaged, can result in increased ion leakage and water loss, and consequently leads to increased mortality. To prevent chilling-induced damage, plants have developed a variety of mechanisms that help to maintain cellular integrity. The Z-3-HAL responsive *COR* genes reported herein are all putative contributors to this protective mechanism in plants. While structurally diverse, *COR* proteins exhibit no enzymatic activity, but rather bind various intra- and extra-cellular structures, thereby enforcing structural integrity during these potentially damaging episodes, resulting in significantly reduced damage.

For example, dehydrins (DEHs) are a class of thermostable hydrophilic proteins that commonly accumulate in plants under drought and cold stress [22,23]. DEHs are typically characterized by a lysine rich K-segment that is highly conserved in plants. DEHs are omnicellular, and are thought to stabilize proteins and membranes in the respective subcellular compartments [22,30,31,32,33]. Proteolipids (PROTs) are proteins that contain a covalently bound lipid moiety, which allows them to associate with membranes or proteins that are fully integrated into a membrane. When induced under cold stress conditions, PROTs may be essential for the protection of membrane systems, by binding to specific lipids in a membrane [34,35], thereby providing structural stability. Late embryogenesis abundant proteins (LEAs) also accumulate in plants and other organisms under water stress conditions, including drought and cold [36]. LEAs, like PROTs, appear to bind preferentially to cytoplasmic facing membrane lipids, and stabilize these structures to prevent protein aggregation [20,24,36,37,38]. Little is known about the role of the adhesive/proline-rich proteins (APRPs) and low temperature inducible proteins (LTIs) in the cold stress response, however, both were found to accumulate in plants under cold stress, strongly suggesting that they have a protective function under these conditions [39,40,41,42,43].

While all of these genes are inducible by GLVs, their rather modest but still significant increase in transcript accumulation, particularly under cold stress conditions, may raise the question of their effectiveness in protecting plants against damage. However, activating many genes with analogous functions, albeit on a relatively low level, may provide a cumulative effect, which then effectively protects the maize plant. It further appears that an early increase in these gene products within 90 min after the onset of treatment is more efficient than an increase at later time points, when plants were already exposed to potentially damaging temperatures for several hours. Together, the results of the conductivity assays, as well as the damage assessment after cold treatments, as presented herein, clearly demonstrate the protectiveness of the response to Z-3-HAL, but probably also to other GLVs. It can therefore be concluded that even the modest accumulation of the transcript for an individual gene can lead to improved protection against stresses, if many genes with analogous functions show similar responses. This will then accumulatively result in a better protection against stresses. Most surprisingly, treatment with Z-3-HAL, even during the cold, provided the same additional protection as what was previously shown for Z-3-HAC, which has never been demonstrated before. This assay is also more likely to reflect the situation in nature, where cold stress damage may cause the release of GLVs from less protected plants, which can then be utilized by neighboring plants to boost their protective responses, even though the cold stress is already in full gear. We have shown here that both the production of GLVs and the perception are fully functional under those conditions. 

Plants also emit GLVs and other volatile organic compounds (VOC) under various other abiotic stresses, including drought [16], salinity [44], extreme temperature [45,46], ozone [47], and UV-B light [48,49,50]. However, to the best of our knowledge, no VOCs other than GLVs are released at significant levels during cold stress damage and have been shown to provide significant protection in this situation [1,46].

To conclude, our findings strongly suggest that certain GLVs may represent an important component in the plant response to cold stress by providing added protection even when already under stress. This further supports our previous notion [1] stating that the functions of GLVs should not be limited to defense responses against biotic stressors, including insect herbivores and necrotrophic pathogens, but must be expanded towards those stresses that can cause physical damage to the plant, including heat, drought, and cold [1,16,46,50]. The findings presented herein support a broader role for these universal volatiles under these premises.

## 4. Materials and Methods

### 4.1. Chemicals

(*Z*)-3-hexen-1-al (Z-3-HAL) was purchased from Bedoukian (Bedoukian Research, Danbury, CT, USA). All of the solvents used were analytical grade.

### 4.2. Plant Materials

Maize (*Zea mays* var. Kandy King) seeds (J. W. Jung Seed Co., Randolf, WI) were grown in ferti-lome Ultimate Potting Mix in a growth chamber under a 12 h photoperiod at 26 °C with 60% relative humidity. The light intensity was set to ca. 150 μmol m^2^ s^−1^. For all of the experiments, we used 10- to 12-day old seedlings at the late V_1_ stage, with the second and third leaf actively growing.

### 4.3. GLV Release during Cold Stress

Previously, we found that cold-damaged maize seedlings primarily released Z-3-HAL in large quantities. However, collection was done at room temperature after the cold treatment. To better study the effects of cold damage on GLV release, we transferred maize seedlings grown under normal conditions into a cold chamber, and placed them under pre-cooled glass cylinders at −5 °C to ensure damage. The glass cylinders were attached to a volatile collection system, as described previously [1]. The controls were kept at room temperature, but otherwise treated similarly. Volatiles, in particular GLVs, were trapped on HayeSep Q80/100-containing filters (Supelco, Bellefonte, Pa) at a flow rate of 200 mL·min^−1^ for 1 h. An analysis of the GLV release was conducted on a Varian model 3900 GC coupled to Varian Saturn 2200 MS equipped with split–splitless capillary injector systems in electron impact mode (EI). The data collection, storage, and subsequent analysis were performed on a computer using the Varian MS Workstation software. Helium at a constant flow velocity of 1 mL/min was used as a carrier gas. The analyses of the volatile collections were performed on a fused silica capillary column (Equity™ 30 m × 0.25 mm inner diameter with a 0.25-µm-thick film of bonded methyl silicone). The GC was programmed as follows: initial temperature 40 °C for 2 min, then temperature programmed at 15 °C/min to 250 °C. All of the injections of were made in the splitless mode. The compounds were identified by comparison to authentic standards (retention time and fragmentation). Dihydro jasmone was used as an internal standard (1 µg per sample).

### 4.4. In-Cold Reatment with Z-3-HAL and Transcript Accumulation for Cold-Stress Related Genes

To test for the effects of Z-3-HAL on cold stress protection during cold episodes (in-cold protection), maize seedlings were first placed in a cold room at 4 °C for 30 min. The plants were then placed under pre-chilled 10-L glass cylinders, and 20 μg of Z-3-HAL dissolved in dichloromethane (1 μg·μL^−1^) was then pipetted onto a cotton ball in the glass cylinder. The control plants were treated with 20 μL of pure dichloromethane. To analyze the changes in transcript accumulation for selected *COR* genes, we treated seedlings with Z-3-HAL for 1.5 h and 3 h, as described above. The second and third leaves were then cut and snap frozen in liquid nitrogen for RNA extraction. The leaves from three seedlings were pooled for one biological replicate, and three replicates were performed for each time point. We extracted the total RNA from 50–70 mg of ground leaf material using the PowerPlant^®^ RNA Isolation Kit containing DNAse (MO BIO Laboratories, Inc., Carlsbad, CA, USA), with the following modifications: The frozen samples were homogenized in 2 mL screw cap tubes containing 0.5 g of Zirmil microbeads and 200 µL of extraction buffer (PR1) for 20 s at 6000 shakes min^−1^ in a Precellys tissue homogenizer (MO BIO Laboratories, Inc., Carlsbad, CA). After this initial homogenization step, we added 800 µL of PR1 and homogenized the samples for an additional 10 s at 6000 shakes min^−1^. The extract was then processed according to the manufacturer’s instructions.

We used the High Capacity cDNA Reverse Transcript Kit (Applied Biosystems, Foster City, CA, USA) to synthesize the cDNA. We performed real-time PCR using the 7300 Real-Time PCR System (Applied Biosystems). The PCR reactions were performed in a 20 µL volume containing 10 µL of Power SYBR^®^ Green PCR Master Mix (Applied Biosystems), 0.2 µM of forward and reverse primer, and the cDNA equivalent to 25 ng of total RNA. The primer specificity was confirmed by melting curve analysis, and the relative transcript levels were calculated using the 2^−ΔΔCT^ method [51], with *Cullin* (*CUL*) as a reference gene [52]. The genes were selected based on previous results [1].

### 4.5. Primer Sequences

*APRP1*: F 5′-CGAAATTTCCTCGCCGTAT, R 5′-CTTTTCCAAGGGTGGTTCAC
*DEH2*: F 5′-GCCATCCCTAATTAAGCCAA, R 5′-CTGGGTGTTCCTCTCATCCT
*LEP1*: F 5′-TGCTCGAGTACGAGATGTGG, R 5′-CTGATCATGTCCCAGACAGC
*LTI*: F 5′-GCAGGTGGAGGATTCTCG, R 5′-GCTGGTCGTCGTAGAAGAGG
*PROT*: F 5′-CTGGGGGTGTTCCTCAAGTA, R 5′-CGGTAGCAAAACACGACTGA
*CUL*: F 5′- GAAGAGCCGCAAAGTTATGG, R 5′-ATGGTAGAAGTGGACGCACC

### 4.6. Conductivity Assays

The genes selected for this study all belong to the *COR* complex, and are essential for the maintenance of structural integrity during cold stress episodes. We therefore performed conductivity assays to test if Z-3-HAL does provide additional protection against cold stress damage by analyzing the ion leakage of leaf discs treated in-cold with Z-3-HAL, and their respective controls. Well-watered two-week-old maize seedlings were used to analyze the ion leakage in cold stressed plants with or without treatment with Z-3-HAL. In-cold treatment was done as described above at 4 °C. The maize seedlings were kept in the cold for 1.5 h, then taken back to RT, and allowed to recover for 30 min. We then cut leaf discs (three from each leaf) with a cork borer #6 from the third and fourth leaf (three per leaf), and washed them three times with distilled water. The leaf discs were then placed on a wet paper towel in a closed petri dish, and put in the freezer at −10 °C for 25 min. The controls for both the Z-3-HAL-treated and control leaf discs from the maize seedlings were handled similarly, but kept at RT. Immediately after being taken out of the freezer, the leaf discs were carefully placed in 10 mL of fresh distilled water, and allowed to sit at RT for 30 min while repeatedly being lightly shaken. After carefully removing the leaf discs from the water to avoid further damage, the ion leakage was measured as the changes in conductivity using an electrical conductivity (EC)/total dissolved solids (TDS) meter (COM-100, HM Digital, Culver City, CA, USA) until stable. The leaf discs were then moved back to the vial, and all of the samples were again placed in the freezer at −10 °C overnight, so as to determine the total ion leakage after being totally frozen. The frozen leaf discs and water were removed from the freezer the next morning, allowed to thaw and to reach RT, and the conductivity was measured again, as described above. The relative conductivity is expressed as the ratio between ion leakage after short cold stress, divided by the total ion leakage after overnight freezing and thawing.

### 4.7. Cold Stress Damage Protection by In-Cold Treatment with Z-3-HAL and Its Effect on Growth Response

To test for the protective properties of in-cold treatment with Z-3-HAL on freezing-induced damage, we treated the maize seedlings as described above in the cold with 20 μg of Z-3-HAL dissolved in dichloromethane (1 μg μL^−1^). The controls were only treated with dichloromethane. The plants were left in the cold chamber overnight (for 16 h) and then placed in the freezer at −5 °C for 30 min. These extended exposures were necessary, as cold stress alone already fortifies the plant tissues against further damage. After 30 min, the plants were removed from the freezer and allowed to grow under normal conditions. Damage assessment was done one day later visually on a scale from 0 (no damage) to 4 (dead), by three independent people in the lab.

Previously, we found that the maize seedlings treated with GLVs showed an enhanced growth response after subsequent cold stress. To test whether in-cold treatment with Z-3-HAL might also alter the growth response of maize seedlings after cold treatment, we first measured the length of the third leaf, which was actively growing at this time, and then treated the seedlings with Z-3-HAC, as described above. The GLV-treated and untreated seedlings were then transferred to a cold chamber at 4 °C overnight (16 h). The control plants were kept at RT under identical light conditions. The next morning, the seedlings were returned to the growth chamber and the length of the third leaf was measured again. We made subsequent measurements at 40 h and 64 h after GLV treatment, and calculated the growth rate for each respective period (cm·h^−1^).

### 4.8. Statistical Analysis

We conducted all of the statistical analyses using JMP Pro 12.1.0 software (SAS Institute, Cary, NC, USA). We confirmed the normality and homogeneity of variance with Shapiro–Wilk and Bartlett tests, respectively, and log transformed data if necessary. The parametric data were compared using t-test (for pairwise comparisons) or one-way ANOVA and Tukey HSD post hoc tests. *P*-values of ≤0.05 were considered statistically significant.
